# Genetic pattern and demographic history of cutlassfish (*Trichiurus nanhaiensis*) in South China Sea by the influence of Pleistocene climatic oscillations

**DOI:** 10.1038/s41598-022-18861-x

**Published:** 2022-08-30

**Authors:** Sui Gu, Yun-Rong Yan, Mu-Rong Yi, Zhi-Sen Luo, Hui Wen, Chang-Ping Jiang, Hung-Du Lin, Xiong-Bo He

**Affiliations:** 1 College of Fisheries, Guangdong Ocean University, No.1 Haida Road, Mazhang District, Zhanjiang, 524088 China; 2 Southern Marine Science and Engineering Guangdong Laboratory (Zhanjiang), Zhanjiang, 524000 China; 3 Guangdong Provincial Engineering and Technology Research Center of Far Sea Fisheries Management and Fishing of South China Sea, Guangdong Ocean University, Zhanjiang, 524088 China; 4 The Affiliated School of National Tainan First Senior High School, No. 1, Sec. 1, Mintzu Rd., Tainan, 701 Taiwan

**Keywords:** Population genetics, Genetic variation, Biodiversity, Genetic markers

## Abstract

*Trichiurus nanhaiensis* is one of the most important commercial fish species in the South China Sea. This study aimed to investigate the level of genetic variation and population genetic structure of *T. nanhaiensis* in the South China Sea for the first time, using 281 individuals collected from seven locations along the coast of mainland China, Taiwan, and Hainan Island. A high level of haplotype diversity and low nucleotide diversity were detected in the mitochondrial DNA cyt *b* gene and nuDNA RYR 3 gene. The overall expected heterozygosity (He = 0.693) among the seven populations ranged from 0.681 to 0.706 in microsatellite DNA data, which revealed high levels of genetic diversity. Significant genetic differentiation was found in Taidong populations in Taiwan, revealing the prevention of gene flow caused by the Kuroshio Current. Two major lineages based on the cyt *b* gene suggested that the Taiwan Strait acted as a geographic barrier for *T. nanhaiensis* during the glacier periods in the late Pleistocene. The Bayesian skyline plot also revealed that population demographic expansion of *T. nanhaiensis* was estimated to have occurred in 0.1 Mya. Our results indicated that all populations of *T. nanhaiensis* had experienced a recent genetic bottleneck following recent expansion based on ABC analysis.

## Introduction

Because of the rise in overfishing, degradation, pollution, and industrialization, understanding the genetic diversity and population structure of economically important marine species is one major crucial objective of species conservation strategies and fisheries management^1^. The population genetic pattern and phylogeographic structure in marine species are contingent on historical factors, including Pleistocene climatic oscillations, glacial vicariance, ocean current systems, and life‐history traits for organisms^2,3^. With a wide distribution, as well as extensive larval and adult dispersal, some marine fish are characterized by weak genetic structure and inferred connectivity^4,5^. Several important studies have revealed that species with broad regions, as expected from larval dispersal, showed extensive gene flow and genetic homogeneity^6^. Furthermore, in many cases, this extensive gene flow and genetic homogeneity might be attributed to fewer barriers to gene flow as well as large population sizes. Many previous molecular studies have shown that many marine fish with high mobility exhibit low genetic structure in the northwestern Pacific, such as in the Chinese beard eel (*Cirrhimuraena chinensis*)^7^ and ponyfish (*Nuchequula mannusella*)^8^. Increasing phylogeographic evidence indicates that cyclical glacial fluctuations shaped the distribution of species, demographic expansions, and genetic divergence of marine fish species. During the Pleistocene glacial-interglacial cycles, the area and configuration of marginal seas in the northwestern Pacific changed dramatically. The emergence of the Taiwan Strait associated with sea-level falls measuring approximately 120–140 m during glacial periods might act as phylogeographic barriers that impede the dispersal of marine fish between both sides of the strait^5,9,10^. Recent mtDNA studies indicate that there are two distinct lineages due to vicariance events in some marine fish, such as in Chinese black sleeper (*Bostrychus sinensis*)^5^, large yellow croaker (*Larimichthys crocea*)^11^, *Lepturacanthus savala*^10^, and yellow seabream (*Acanthopagrus latus*)^12^. Understanding the genetic characteristics of marine fish, including their genetic diversity, genetic structure, and population historical demography, provides reference opinions for the long-term management and protection of species germplasm resources^13^.

Cutlassfishes (of the family Trichiuridae) belonging to the suborder Scombroidei of Perciformes is a diverse group of demersal fishes, including three subfamilies, eight genera, and twelve species in mainland China. The fisheries of cutlassfishes showed declining trends over recent years on the coast of mainland China and the third most marine catches in the South China Sea (SCS). *Trichiurus nanhaiensis*, Wang and Xu 1992 featuring a very elongated and highly compressed body with a deeply forked caudal fin, is a benthic fish of the family Trichiuridae that inhabit the continental shelves and shallow seas (20–200 m) of tropical and temperate waters, and is wide geographical distribution in the Indo-Pacific Ocean, including the South China Sea. The Taiwan Strait is thought to be the northernmost limit of its range in the Western Pacific. *Trichiurus nanhaiensis* is one of the most important commercial fish species in the South China Sea, accounting for 13.7–14.2% of the total annual fishing catch in the South China Sea between 2019 and 2020. Previous studies on *T. nanhaiensis* have focused mainly on the mitogenome taxonomy^14^, DNA barcoding^15^, characterization of microsatellite DNA^16^, reproduction^17^ and demographic history^18,19^. Understanding the genetic structure of *T. nanhaiensis* populations is necessary and is required to formulate strategies for the effective protection and utilization of marine fishery resources^20^. In addition, previous population genetic studies of *T. nanhaiensis* using limited genetic data were used to delineate population structure, and discrepancies among studies have to some extent hindered the effectiveness of fisheries management and conservation^18,19^.

Nuclear DNA is inherited from two parents, and recombination of genes from the parents in every generation, whereas mtDNA is haploid and only maternally inherited, reflecting changes in population structure faster due to its lower effective population size and therefore rapid coalescence time. Microsatellites are simple repetitive sequences and codominant throughout the eukaryote nuclear genome, showing high resolution at the population level^21^. Direct comparisons among mtDNA, nuDNA and microsatellite loci can be very informative for population diversity and genetic structure, as they provide valuable information for evaluation and conservation issues. This study aimed to investigate the level of genetic variation and population genetic structure of *T. nanhaiensis* in the South China Sea (including mainland China, Taiwan, and Hainan Island) for the first time, and it used mitochondrial, nuclear, and microsatellite DNA markers to reveal the distribution of genetic variation in *T. nanhaiensis*. Additionally, our results provide a workable and responsible stock for scientific and conservation purposes to maintain long-term sustainability and resilience to the fishing pressure of *T. nanhaiensis* resources.

## Results

### Mitochondrial and nuclear DNA

**Figure 1:**
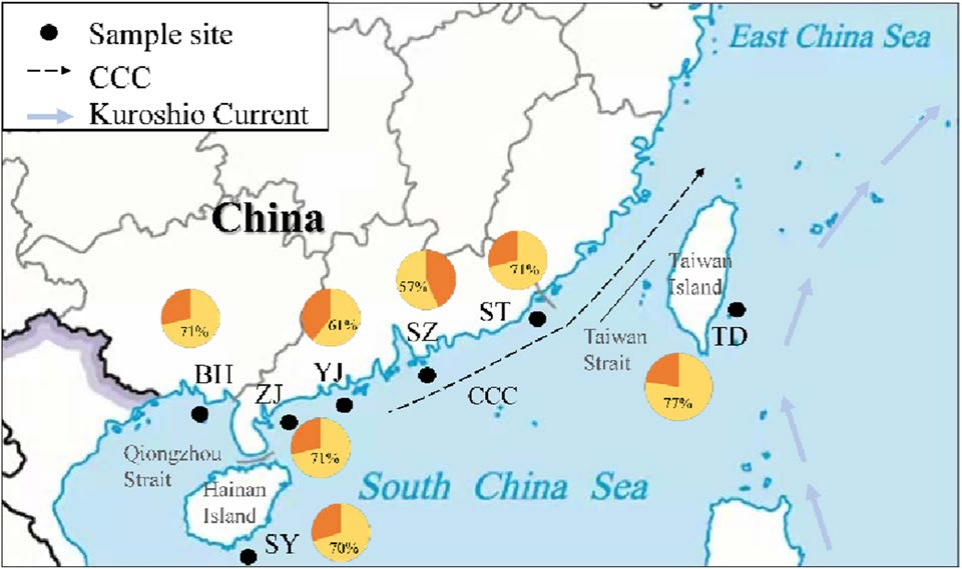
Map of the coast of mainland China illustrating the sampling locations of *Trichiurus nanhaiensis*. Each color in the pie charts represents the frequencies of the private (orange) and shared (yellow) haplotypes in each population. *CCC* China Coastal Current. The sampling site/population code is described in Table 1.

A total of 181 and 57 haplotypes were identified by sequencing 1141 bp and 882 bp of the complete mtDNA cyt *b* gene and nuclear RYR 3 gene from 281 *T. nanhaiensis* individuals, respectively (Table 1, Fig. 1). The most common haplotypes of the mtDNA cyt *b* gene (Hm8, Hm18, and Hm27) (Supplementary Table S1) and nuclear RYR 3 gene (Hn1, Hn2, Hn6, and Hn8) were shared by specimens from seven and all populations, respectively (Supplementary Table S2). The average haplotype diversity of the mtDNA cyt *b* gene was high (0.989), ranging from 0.981 (ST) to 0.995 (ZJ), and the average nucleotide diversity (θ_π_) was low (0.005), ranging from 0.004 (ZJ) to 0.005 (YJ) (Table 1). The average haplotype diversity of the nuDNA RYR 3 gene was high (0.724), ranging from 0.650 (SZ) to 0.812 (SY), and the average nucleotide diversity (θ_π_) was low (0.001), ranging from 0.001 (SZ) to 0.002 (SY) (Table 1).

**Table 1:** Samples used for analysis, location, code and summary statistics.

Population	Code	Coordinares	Sample size	mtDNA				nuDNA				Microsatellite					
				N of h	H	θ_π_ (%)	θ_ω_ (%)	N of h	H	θ_π_ (%)	θ_ω_ (%)	A_a_	A_R_	H_O_	H_E_	F_IS_	HWE
**Mainland China**																	
1. Shantou	ST	23.02° N 116.93° E	63	45	0.981	0.451	0.987	16	0.740	0.149	0.398	10.6	7.789	0.563	0.685	0.172	0.160
2. Shenzhen	SZ	21.86° N 114.15° E	40	30	0.985	0.434	0.867	11	0.650	0.100	0.183	9.4	7.907	0.601	0.690	0.109	0.274
3. Yangjiang	YJ	21.31° N 112.19° E	36	33	0.994	0.528	0.889	10	0.680	0.127	0.211	8.5	7.537	0.614	0.685	0.100	0.422
4. Zhanjiang	ZJ	20.90° N 110.88° E	30	28	0.995	0.381	0.665	13	0.702	0.129	0.195	8.2	7.672	0.607	0.702	0.131	0.352
5. Beihai	BH	21.23° N 109.24° E	39	34	0.988	0.422	0.851	13	0.697	0.123	0.253	9.4	8.113	0.628	0.706	0.103	0.185
**Taiwan Island**																	
6. Taidong	TD	22.72° N 121.23° E	25	22	0.990	0.511	0.698	12	0.693	0.123	0.202	8.5	8.453	0.555	0.702	0.191	0.189
**Hainan Island**																	
7. Sanya	SY	17.89° N 109.60° E	48	40	0.988	0.475	1.029	25	0.812	0.185	0.419	8.8	7.309	0.590	0.681	0.127	0.245
Mean					0.9893	0.465	1.724		0.724	0.139	0.673	9.1	7.826	0.594	0.693		

^a^Number of haplotypes; ^b^Haplotype diversity; ^c,d^Nucleotide diversity; ^e^Average number of alleles; ^f^Mean allelic richness; ^g^Mean observed heterozygosity; ^h^Mean expected heterozygosity; ^i^Mean level of inbreeding observed; ^j^Hardy–Weinberg equilibrium.

The pairwise *F*_ST_ values ranged from -0.011 (between SY and YJ) to 0.084 (between TD and SZ), with a mean value of 0.023 in the mtDNA cyt *b* gene. The pairwise *F*_ST_ values between the populations from TD and other populations were differentiated, except for the YJ population in the mtDNA cyt *b* gene (Supplementary Table S3). The pairwise *F*_ST_ values ranged from 0.001 (between ST and BH) to 0.060 (between YJ and ZJ), with a mean value of 0.020 in the nuDNA RYR 3 gene. No significant correlation was found between genetic distance and geographic distance by using IBD analyses (r = 0.438, p = 0.087).

**Figure 2:**
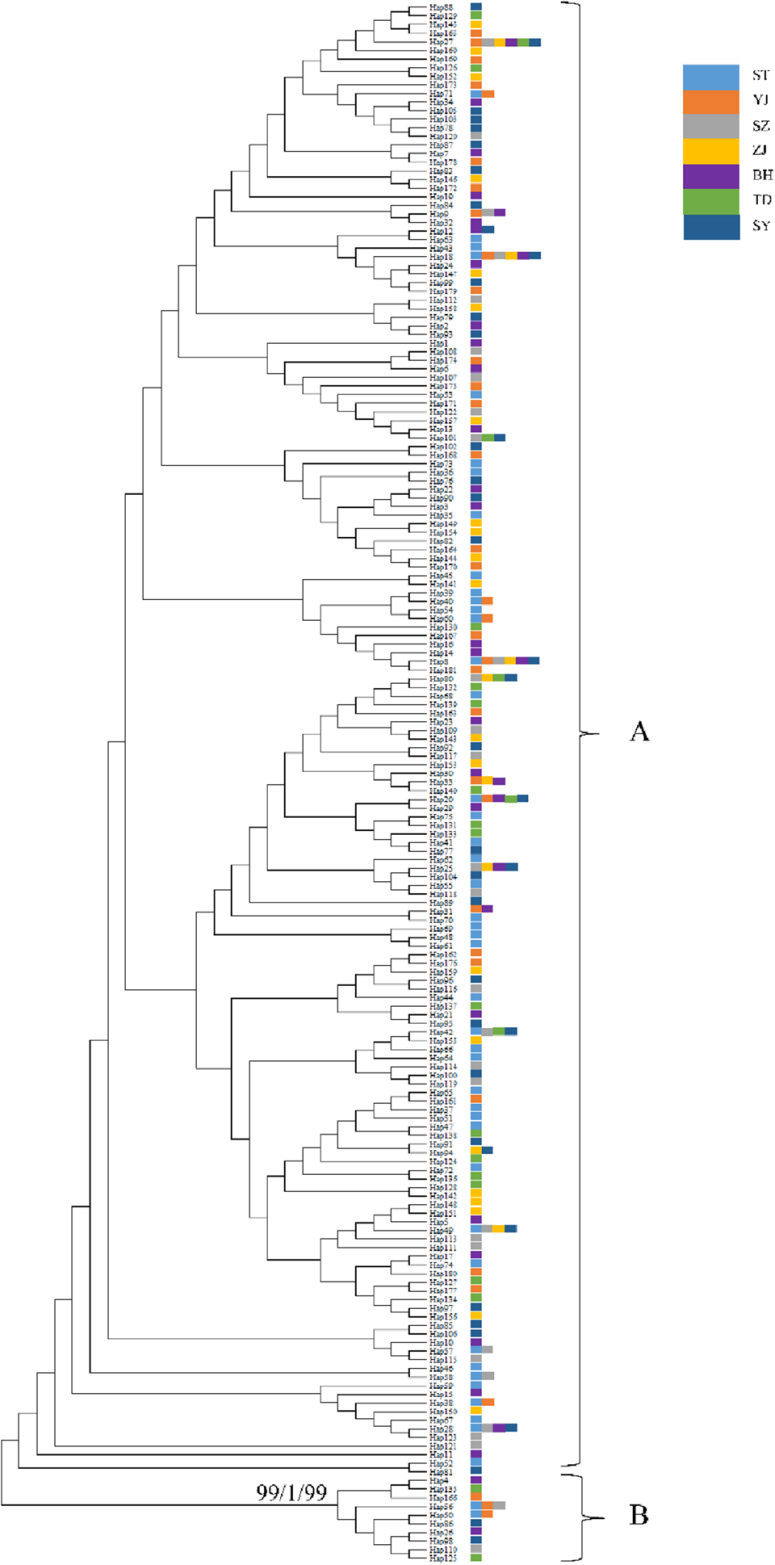
Phylogenetic trees reconstructed from mitochondrial sequences of the cyt *b* gene in *Trichiurus nanhaiensis*. The values above the branches are bootstrap values for the NJ, BI, and ML analyses.

**Figure 3:**
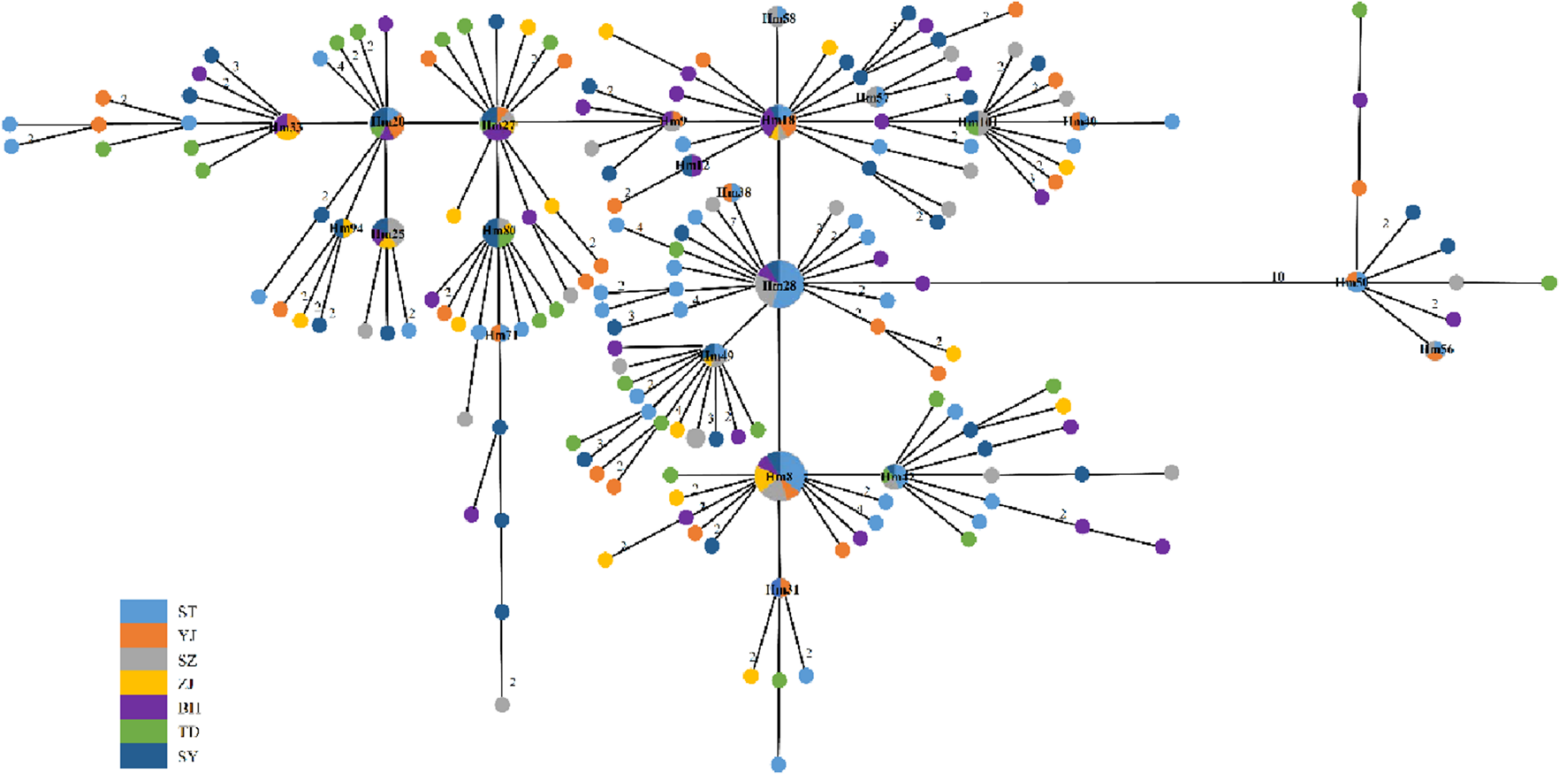
The minimum-spanning haplotype network inferred from the mitochondrial cyt *b* gene of *Trichiurus nanhaiensis* from seven geographic samples in mainland China. Each circle indicates each haplotype, and the size of each circle is related to its haplotype distribution frequency. Each color in haplotype network circles represents each geographic sample.

**Figure 4:**
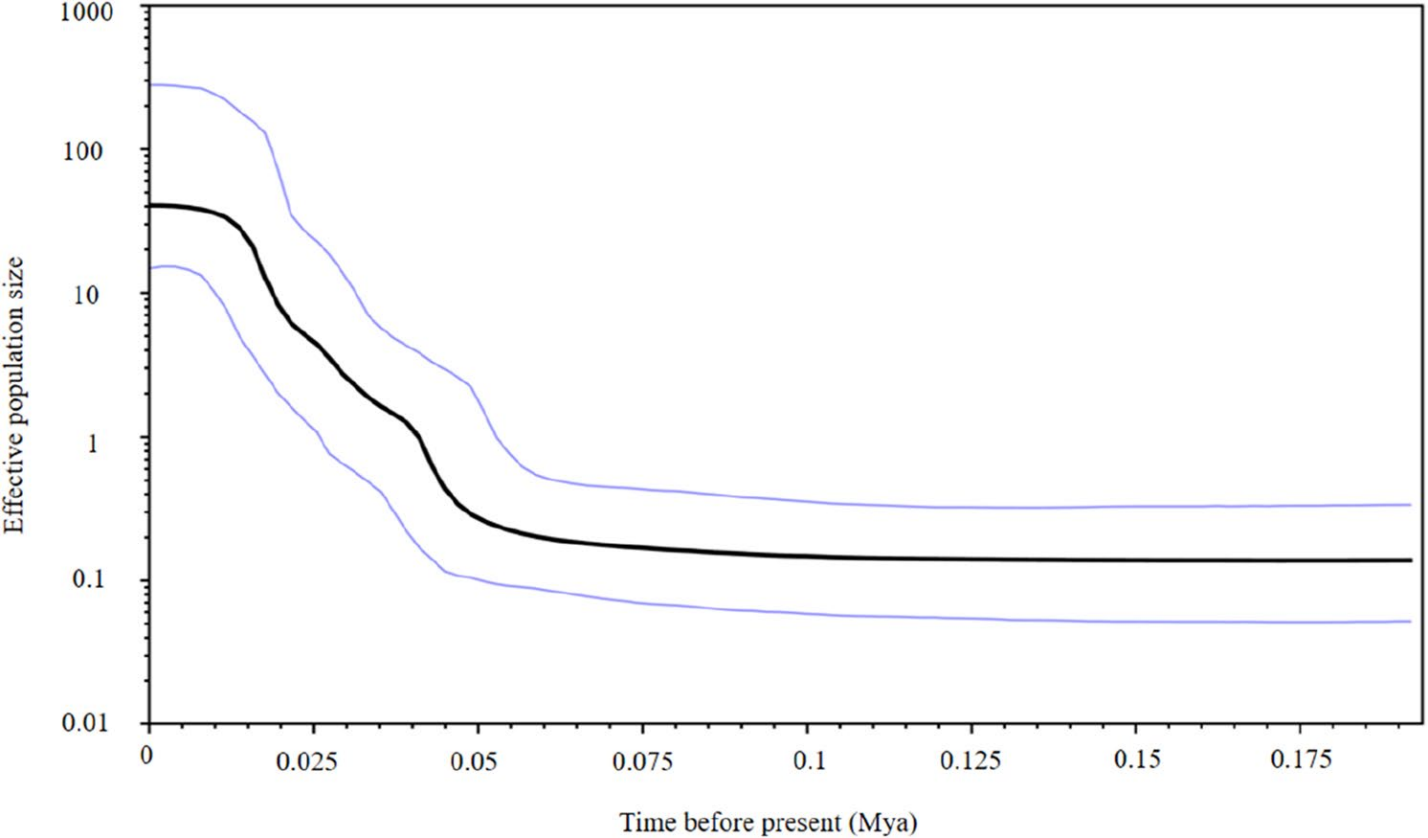
Bayesian skyline plot of the effective population sizes over time for *Trichiurus nanhaiensis*. The y-axis is the product of effective population size (Ne) and generation length on a log scale, while the x-axis is the time scale before present in units of million years ago.

Hierarchical analyses of molecular variance (AMOVA) from the probable factors shaping genetic structure demonstrated that most of the genetic variation was among individuals within populations in the mtDNA cyt *b* gene and nuclear RYR 3 gene, i.e., one group (97.79%, 98.42%), two groups (Scenario I, 94.47%, 97.46%), two groups (Scenario II, 99.00%, 98.74%), and three groups (Scenario III, 97.30%, 98.32%) (Table 2). However, only 2.21%, 4.19%, 1.87%, and 0.97% of the total variation was found among the groups in one group, two groups (Scenario I), two groups (Scenario II), and three groups (Scenario III) in the mtDNA cyt *b* gene, respectively (Table 2). Phylogenetic trees and haplotype networks based on the cyt *b* gene support the formation of two major lineages, and genetic clusters do not correspond to geographical sampling sites (Figs. 2, 3). However, the phylogenetic relationship and networks based on the nuDNA fragments showed no evidence of significant geographical structure corresponding to the sampling locations (Supplementary Figs. S1, S2). Neutrality tests, mismatch distribution analysis, and Bayesian skyline plots were used to analyze signatures of historical demographic events. The significant negative values of Tajima's D and Fu's *F*s tests demonstrated deviation from the neutrality test, suggesting past population expansion for *T. nanhaiensis* (Tajima's D, − 2.221, *P*  < 0.001 and Fu's *F*s, − 358.690, *P* < 0.000). Likewise, the same results were supported by the presence of a star-like topology in the cyt *b* gene tree and by the results of mismatch distribution analysis, which showed unimodal distributions (Supplementary Fig. S3). The Bayesian skyline plot showed that *T. nanhaiensis* populations seem to have experienced demographic expansion starting approximately 100,000 years ago and a flat population history during the Pleistocene (Fig. 4). The current (θω) and historical (θπ) genetic diversity was 0.017 and 0.0046, respectively. When the current genetic diversity was larger than the historical genetic diversity, the *T. nanhaiensis* populations revealed a pattern of decline^22^.

**Table 2:** Analysis of molecular variance (AMOVA) for *Trichiurus nanhaiensis* populations based on three molecular markers.

Scheme	Category description	mtDNA			nuDNA			microsatellite		
		Variance components	Percentage variation	P	Variance components	Percentage variation	P	Variance components	Percentage variation	P
**1. One group**										
	Among populations	0.0588	2.21	**0.0000**	0.0097	1.58	**0.0000**	0.0088	0.20	**0.0000**
	Within populations within groups	2.5985	97.79	**0.0000**	0.6056	98.42	**0.0000**	0.5889	13.38	1.0000
	Within individuals							3.8047	86.42	**0.0000**
**2. Two groups (TD) (ST, SZ, YJ, ZJ, BH, SY) [divided by the Taiwan Strait]**										
	Among groups	0.1153	4.19	**0.0000**	0.0074	1.20	**0.0000**	0.0323	0.73	0.1320
	Among populations within groups	0.0367	1.33	**0.0000**	0.0083	1.34	**0.0020**	0.0026	0.06	0.3011
	Among individuals within populations	2.5985	94.47	**0.0039**	0.6056	97.46	0.1388	0.5889	13.30	**0.0000**
	Within individuals							3.8047	85.92	**0.0000**
**3. Two groups (SY) (ST, SZ, YJ, ZJ, BH, TD) [divided by the Qiongzhou Strait]**										
	Among groups	− 0.0492	− 1.87	**0.0000**	− 0.0031	− 0.50	**0.0000**	0.0027	0.06	0.4282
	Among populations within groups Groups 13.37 86.39	0.0753	2.87	**0.0000**	0.0108	1.76	**0.0000**	0.0079	0.18	0.1202
	Among individuals within populations	2.5985	99.00	0.9609	0.6056	98.74	0.6305	0.5889	13.37	**0.0000**
	Within individuals							3.8047	86.39	**0.0000**
**4. Three groups: (mainland) (Taiwan) (Hainan)**										
	Among groups	0.0260	0.97	**0.0000**	0.0011	0.18	**0.0000**	0.0163	0.37	**0.0000**
	Among populations within groups	0.0461	1.73	**0.0010**	0.0092	1.49	**0.0010**	0.0008	0.02	**0.0000**
	Within populations	2.5985	97.30	0.1926	0.6056	98.32	0.3891	0.5889	13.35	0.4233
	Within individuals							3.8047	86.26	0.1320

Significant scores (*P* < 0.05) are shown in bold face.

### Microsatellite DNA

No indication of genotyping errors due to stutter bands, large allele dropout, or null alleles was revealed using a microchecker^23^. A total of 118 alleles were detected for the 13 polymorphic markers, and the number of alleles per marker ranged from 3 to 27, with an average of 9.0, in 281 individuals, with 72 unique alleles in the total population. The sample size, number of alleles (*N*_a_), number of allelic richness (*A*_R_) per population, expected heterozygosity (*H*_E_), observed heterozygosity (*H*_O_), inbreeding coefficient (*F*_IS_), and deviation from Hardy–Weinberg equilibrium (HWE) for each population are shown in Table 1. The average number of alleles and allelic richness for each population ranged from 8.200 (ZJ) to 10.600 (ST) (average = 9.050) and from 7.530 (YJ) to 8.450 (TD) (average = 7.820), respectively. The mean observed and expected heterozygosity was 0.590 and 0.690, ranging from 0.555 (TD) to 0.628 (BH) and from 0.681 (SY) to 0.706 (BH), respectively. The fixation index (*F*_IS_) in all populations was positive, indicating that the heterozygote deficiencies ranged from 0.100 (YJ) to 0.191 (TD) (Table 1).

The average number of alleles (*N*_a_) and allelic richness (*A*_R_) per locus were 9.0 and 7.8, ranging from 3 (Tna3) to 27 (Tna16) and from 2.771 (Tna3) to 22.406 (Tna16), respectively. The observed heterozygosity (*H*_O_) and the expected heterozygosity (*H*_E_) were 0.594 and 0.693, ranging from 0.244 (Tna1) to 0.759 (Tna14), and ranging from 0.343 (Tna1) to 0.951 (Tna16), respectively (Supplementary Table S4). The average value of *F*_IS_ was 0.145, and most microsatellite DNA loci showed a significant heterozygote deficit and positive *F*_IS_ values, except Locus Tna42 (− 0.017). The characteristics of all 13 microsatellite loci in *T. nanhaiensis* are shown in Supplementary Table S4. The pairwise *F*_ST_ values, with an average of 0.042(10^–2^), ranged from − 0.002 (between ZJ and YJ) to 0.014 (between SY and TD), and the *R*_ST_ values, with an average of 0.009, ranged from − 0.014 (between TD and SZ) to 0.040 (between ZJ and TD) (Supplementary Table S5). The pairwise *F*_ST_ values between the TD population in Taiwan and other populations were relatively large in all pairwise comparisons, and differentiation between ST, ZJ, BH, and SY was significant (Supplementary Table S5). The results showed a nonsignificant correlation within IBD analyses between genetic differentiation and geographic distance using microsatellite DNA data (r = 0.618, p = 0.031). AMOVA showed that most of the genetic diversity was within individuals, i.e., one group (86.42%), two groups (Scenario I, 85.92%), two groups (Scenario II, 86.39%), and three groups (Scenario III, 86.26%) (Table 2). When the populations were divided into two groups (Scenario I), two groups (Scenario II), and three groups (Scenario III) according to the geographical barriers, only 0.73%, 0.06%, and 0.37% of the total variation was found among the groups, respectively.

**Figure 5:**
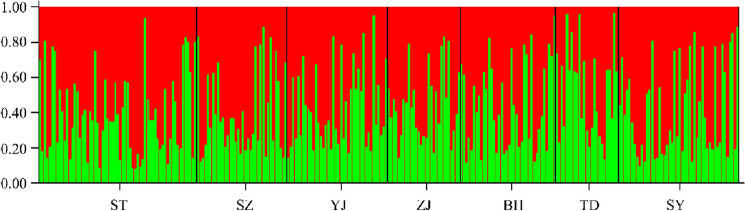
Clustering of individuals by structure at K = 2. Individuals are represented by vertical bars. Each vertical column represents one individual, and the separation of the column into two colors represents the estimated probability of belonging to one population or the other. Different colors in the same individual indicate the percentage of the genome shared with each cluster according to the admixture proportions. The y-axis represents the probability of belonging to a certain cluster, while each population (code name given in Table 1) delimited by a black solid vertical line is reported on the x-axis.

**Figure 6:**
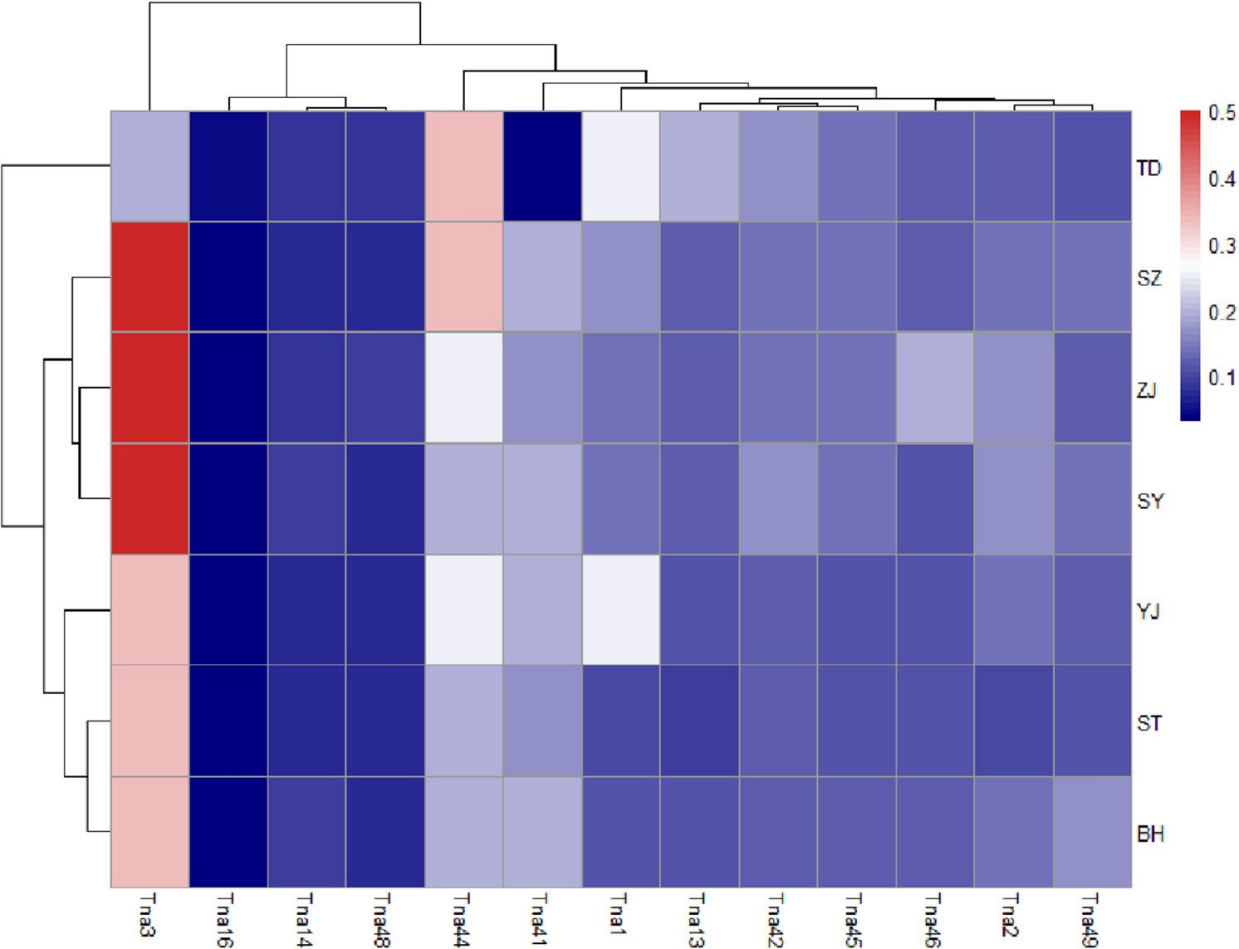
Heat map representing allele frequency for 13 loci in all 7 population as the gradual increase in color density from lighter color (blue) to darker color (red) means the increase in the allele frequency as the red color, which indicates the highest value of allele frequency.

**Figure 7:**
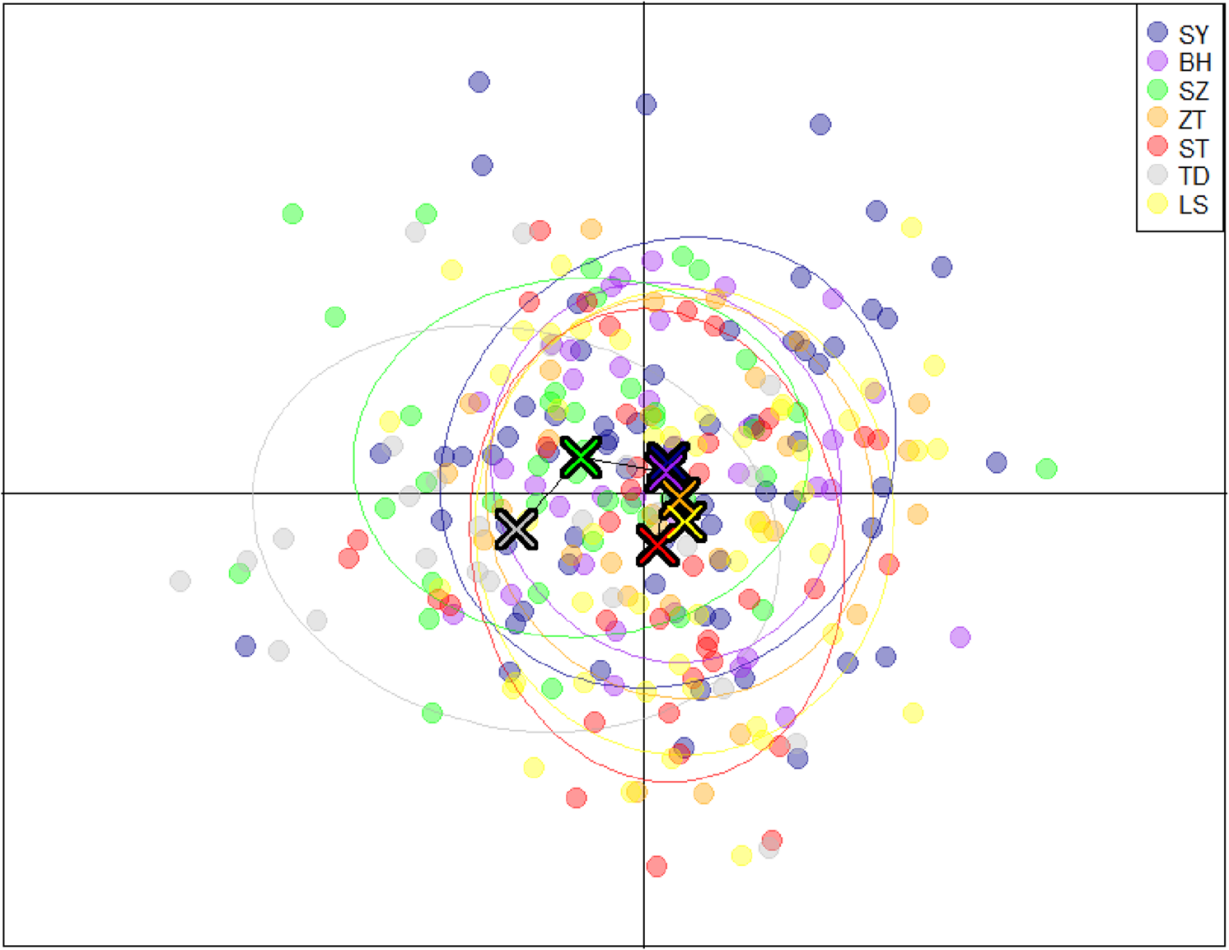
Discriminant analysis of principal components (DAPC), showing relationships among six populations of *Trichiurus nanhaiensis* clusters based on thirteen microsatellite loci *LYG* Lianyungang, *ND* Ningde, *RP* Raoping, *ZH* Zhuhai, *ZJ* Zhanjiang, *BH* Beihai. See Table 1 for the sampling sites/population codes.

We used the Bayesian model-based clustering algorithm implemented in STRUCTURE 2.3.3 software to assign individuals into populations by estimated individual admixture proportions and to explore different numbers of populations K to the population structure based on microsatellite data. The most likely number of clusters was determined by Structure Harvester, which indicated that all seven populations were separated into two distinct genetic clusters (K = 2, Ln P(K) = − 172.360; Stdev LnP(K) = 27.976; Delta K = 6.036) (Supplementary Table S6). Although the results of the STRUCTURE analysis supported K = 2, all sampled individuals exhibited admixture among two clusters with roughly equal contributions (Fig. 5). Microsatellite DNA data were used to provide a platform for data visualization and construction of a heatmap representing the allele frequency for all 13 loci in the 7 populations, as presented in Fig. 6. The interpretation of the color intensity was as follows: the relationship between the color and the allele frequency is given by the color bar on the right. The firebrick color indicates the highest value of allele frequency. The heatmap of allele distribution and frequency across populations for each locus showed populations TD clustered at the top of the heatmap, while the remaining populations belonged to the other group. The results of DAPC analyses indicated that the populations of *T. nanhaiensis* could be split into two groups (Fig. 7). Populations TD belonged to one group, and the remaining populations belonged to the other group.

### Approximate Bayesian computation

**Figure 8:**
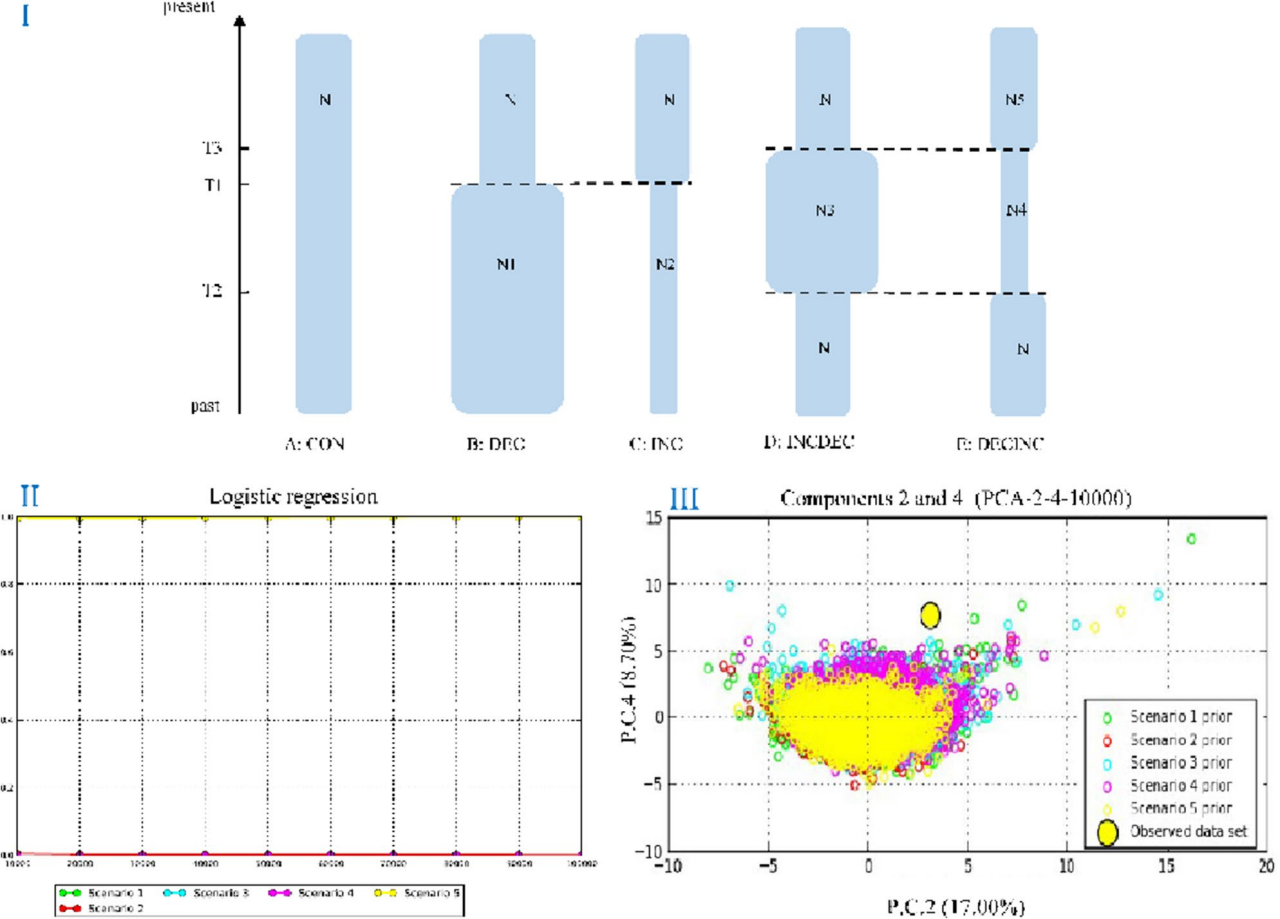
(I) Schematic representation of five demographic scenarios for *Trichiurus nanhaiensis* tested by approximate Bayesian computation (ABC). Time and effective population size are not to scale. (II) The results of a logistical model comparing posterior probability of each scenario with the number of simulations used to calculate it. (III) The prior distribution of parameters corresponding to the five scenarios tested. Colors reflect each scenario.

Among the five different plausible demographic scenarios (see Fig. 8I) tested in our DIYABC simulations, Scenario 5 (DECINC model) had the highest PP and 95% CI (posterior probability = 0.998 [0.995,1.000]) compared with the other scenarios. The results of a logistical model comparing posterior probability of each scenario with the number of simulations used to calculate it (see Fig. 8II). The prior distribution of parameters corresponding to the five scenarios tested (see Fig. 8III). This scenario indicated that *T. nanhaiensis* experienced a reduction in the effective population size in the past, followed by a single instantaneous increase in population size.

## Discussion

### Genetic diversity

Monitoring genetic diversity is an integral part of conservation biology, and maximizing the maintenance of genetic variation is necessary to dissect the integrated effects of evolutionary and ecological processes following environmental changes^24^. Here, we provided an assessment of molecular markers (mitochondrial, nuclear, and microsatellite DNA) to analyze the genetic diversity of *T. nanhaiensis*, which is of importance to biodiversity conservation and the sustainable use of biological resources. High haplotype diversity (*h* = 0.989) and low nucleotide diversity (0.005) were found in *T. nanhaiensis* populations based on the mitochondrial cyt *b* gene. High haplotype diversity was similar to the haplotype diversity of most marine fishes on the coastlines of mainland China, suggesting large effective population sizes over time [e.g., *Pampus argenteus*^25^ (*h* = 0.736); *Terapon jarbua*^26^ (*h* = 0.857); *Acanthogobius ommaturus*^27^ (*h* = 0.923); *Scatophagus argus*^28^ (*h* = 0.870)]. The nucleotide diversity of *T. nanhaiensis* was higher than the nucleotide diversity of *L. savala*^8^ but lower than the nucleotide diversity of *T. japonicas* in the family Trichiuridae along the coastlines of mainland China^18^. Population size is considered to be an important factor in the maintenance of genetic variation, and small populations should show lower levels of genetic variability than large populations^29^. The current effective population sizes of *T. japonicas*, the dominant cutlassfish species, were larger than the current effective population sizes of *T. nanhaiensis* throughout Taiwan and the coast of mainland China^15,18,19,30^.

The values of the average number of alleles per population (9.0) of *T. nanhaiensis* were relatively lower than the average number of alleles per population of the relative species *L. savala* and other marine fish populations along the coast of mainland China (e.g., *N. albiflora N*_a_ = 12.020^31^; *Lateolabrax maculatus N*_a_ = 28.727^32^; *Larimichthys polyactis N*_a_ = 24.950^33^). In this study, the average value of expected heterozygosity (0.693) of *T. nanhaiensis* was relatively lower than the average value of expected heterozygosity of marine fish (average *H*_E_ = 0.790) with many marine fish populations^34^. In this study, the positive inbreeding coefficient (*F*_IS_ = 0.145) of *T. nanhaiensis* indicates heterozygosity deficiency in all populations, which might be attributed to inbreeding. Previous studies have reported that low observed heterozygosity points to a deficiency of heterozygotes and high levels of inbreeding in the waters off mainland China (e.g., *Larimichthys polyactis*^33^; *Collichtys lucidus*^35^; *Lateolabrax maculatus*^32^), as well as in other related species (e.g., *L. savala*^10^). In addition, the loss of genetic diversity may reflect past historical events associated with current evolutionary forces, such as fishing pressure or habitat loss^19^. Loss of genetic diversity is most often associated with reduced population adaptive capacity fitness through inbreeding depression, the maintenance of the high levels of genetic variability in natural populations has been recommended for conservation.

### Phylogeography and population structure of *Trichiurus nanhaiensis*

Many previous studies suggested that the major drop in sea level in the Taiwan Strait acted as a biogeographic barrier during major falls in sea level in the Pleistocene and might have cut off migration on either side of the strait and presented two lineages in marine fishes, such as the genera *Helice*^36^, *B. sinensis*^9^, and *L. savala*^10^. Our results revealed that haplotype networks and phylogenetic trees support the formation of two major lineages based on the cyt *b* gene, and genetic clusters do not correspond to geographical groups. During the Pleistocene, the East China Sea and the South China Sea were semienclosed marginal seas and separated by the Taiwan Strait. We suggested that the Taiwan Strait acted as a geographic barrier for *T. nanhaiensis* during the glacier periods in the late Pleistocene and gene flow due to the absence of physical barriers during glacial retreat, while more recent secondary contacts could have played a major role^37^. The results of the AMOVA test based on mitochondrial, nuclear, and microsatellite DNA data indicated that there were no significant differences at all hierarchical levels. All genetic markers, including mitochondrial, nuclear, and microsatellite markers, congruently showed a similar genetic pattern, revealing overall low genetic differentiation among *T. nanhaiensis* populations, by previous studies on other marine fishes among the geographical stocks in the South China Sea (e.g., *L. savala*^10^; *Nemipterus bathybius*^38^). The results from STRUCTURE indicated the presence of two clusters after independent clustering of all populations based on microsatellite DNA data, which may be explained by the high dispersal capacity of *T. nanhaiensis*. The scenario of evolutionary diversification of lineages followed by postglacial expansion facilitating secondary contacts was observed in previous studies (e.g., *S. macrorhynchos*^39^; *Scatophagus argus*^28^; *L. savala*^10^). A previous study in *Trichiurus* species suggests a genetic well mixture and a lack of distribution barrier for *T. nanhaiensis*^17^. Ocean currents, which change with the monsoon and are highly variable in the SCS, play an important role in transporting eggs, larvae, or juveniles of *T. nanhaiensis*^40,41^. We suggested that the high dispersal ability of marine fish may be appeared by gene flow following divergence, indicating that divergent mitochondrial lineages arose within a panmictic population. The results from DAPC and the heatmap of allele distribution and frequency across populations revealed that population TD was distinctly separated from the other geographic populations. Population TD was located in southern Taiwan, and samples were collected from the Pacific Ocean. We suggest that population TD belonging to peripheral populations may always have low genetic variability due to isolation and founder effects, but the differentiation of the populations is high owing to genetic drift and reduced gene flow under the influence of the Kuroshio Currents^42^.

### Demographic history and DIY-ABC

Both mismatch distribution analyses and neutrality indices of Tajima’s *D* and Fu’s *F*s tests suggested that *T. nanhaiensis* populations indicate recent demographic expansion. Moreover, the star-like structure of the haplotype network shows consistent evidence for past recent population bottlenecks followed by population expansion. The Bayesian skyline plot also revealed that population demographic expansion of *T. nanhaiensis* was estimated to have occurred in 0.1 Mya. The presence of a consistent signal of recent population expansion gives a similar estimated expansion time to other previous studies in *T. nanhaiensis*, which suggests that the population expansion event took place in the late Pleistocene^18,19^. Furthermore, the results of our comparison to the previous study of marine fish in the South China Sea thus provide considerable evidence of population growth and expansion in *T. nanhaiensis* (e.g., *S. macrorhynchos*^39^; *Tridacna maxima*^43^; *Scatophagus argus*^28^; *Nemipterus bathybius*^38^; *L. savala*^10^). However, a higher θω than θπ indicated population decline, and all seven populations appeared to experience genetic bottlenecks based on the microsatellite markers in *T. nanhaiensis*. The demographic scenario of *T. nanhaiensis* illustrates an even more complex history. Our best-fitting ABC model [Scenario 5 (DECINC model)] was identified with recent expansion preceded by a bottleneck. Our results support previous studies showing that the population of *T. nanhaiensis* steadily declined during 45–165 kya and exhibited a pattern of glacial growth^18^.

## Materials and methods

### Sample collection, DNA extraction, and microsatellite genotyping

A total of 281 *T. nanhaiensis* were collected from seven locations in the South China Sea in 2019 (Table 1, Fig. 1). All studies in animals were conducted by ethical committee guidelines and approved by the Animal Research and Ethics Committee of College of Fisheries, Guangdong Ocean University (China), and the study was carried out in compliance with the ARRIVE guidelines. Fin tissues were stored in 95% alcohol for DNA extraction after morphological identification of all samples. Total DNA was extracted using a DNA extraction kit (Sangon Biotech, Shanghai, China).

The mitochondrial cytochrome *b* (Cyt *b*) gene was amplified using the gsCY4-F (5′-TCCCTCATTGACCTTCCA-3′)/gsCY4-R (5′-GGTTGCGGTTCAGTTGAG-3′) primer pair, and the nuclear DNA gene (its protein-coding sequence) for ryanodine receptor 3 (RyR3) was amplified using the primers RYR3-F22 (5′-TCGGTAAGCARATGGTGGACA-3′) and RYR3-R931 (5′-AGAATCCRGTGAAGAGCATCCA-3′). The 13 microsatellite loci were referenced from a previous study^14^. PCRs were performed in 25 μl of the total volume containing 3 μl genomic DNA, 12.5 μl Taq mix, 1 μl primer, and 7.5 μl ddH_2_O. PCR amplification was performed in a Biometra thermal cycler under the following conditions: 94 °C for 5 min for initial denaturation, followed by 33 cycles of 92 °C for 0.5 min, 57 °C for 1 min, extension at 72 °C for 1 min, and a final extension at 72 °C for 10 min. All PCR products were purified and sequenced commercially (Sangon Biotech, Shanghai, China). Electrophoresis and genotyping were conducted by an ABI PRISM 3730 Genetic Analyzer automated DNA sequencer (Applied Biosystems, Inc., Foster City, CA, USA) and GeneMapper 4.0 (Applied Biosystems). All nucleotide sequences were deposited in GenBank (accession numbers: OM303509-OM303789 for Cyt *b* and OM388568-OM389129 for RYR3).

### Data analysis

The Cyt *b* and RyR 3 sequence alignments for all 281 samples were separately performed using Clustal X 2.0 software^44^. The PHASE algorithm in DnaSP v5.0 software^45^ was used to resolve the haplotype phases of RyR3 sequences that contained more than one ambiguous site and analyze summary statistics such as the number of haplotypes (Nh), haplotype diversity (*h*), and nucleotide diversity (θ_π_ and θ_ω_)^22^. We constructed neighbor-joining (NL) trees using MEGA X^46^ to analyze the phylogenetic relationship of *T. nanhaiensis*. Bayesian inference (BI) and maximum likelihood (ML) were performed using PhyloSuite v1.2.2^47^. The best-fit partitioning strategy and nucleotide substitution model for BI and ML tree were selected by ModelFinder installed in Phylosuite v1.2.2 with the greedy algorithm and BIC (Bayesian information criterion) criteria. Haplotype networks for Cyt *b* and RyR 3 were computed using the minimum-spanning network method (minspnet in Arlequin 3.5)^48^. In addition, the neutrality test (Tajima’s D^49^ and Fu’s Fu test^50^) and mismatch distributions were evaluated to test the hypothesis of demographic expansion using DnaSP v5.0 software^45^. Bayesian skyline plots (BSPs) were implemented in BEAST v1.8.2 for *T. nanhaiensis* to determine the effective population size changes over time^51^. The MCMC chains were run three times each for 100 million generations, sampling every 10,000th generation for each data set. In the BSPs analysis, the effective sample size (ESS) values for each parameter was larger than 200, and the 95% highest posterior density (HPD) intervals were reported. Bayesian skyline plots (BSPs) were analyzed and drawn using the tracer v1.6 program^52^. A 2.03%/MY divergence rate has been calibrated for the mtDNA cyt *b* genes in *Trichiurus* fishes for population expansion^18^.

Pairwise *F*_ST_ between all pairs of populations and analysis of molecular variance (AMOVA) were executed in Arlequin 3.5^48^. Seven populations were grouped with three scenarios based on geographical barriers: (1) Scenario I: two independent groups divided by the Taiwan Strait (TD; ST, SZ, YJ, ZJ, BH, SY); (2) Scenario II: two independent groups divided by the Qiongzhou Strait (BH; ST, SZ, YJ, ZJ, SY, TD); (3) Scenario III: three independent groups that included the mainland group, the Taiwan island group and the Hainan island group (ST, SZ, YJ, ZJ; TD; SY, BH). In addition, an isolation-by-distance Mantel test was performed to examine correlations between genetic differentiation and geographic distance by IBD 1.52 for Windows^53^. The Google Earth database (http://earth.google.com) was used to measure the geographic distance in kilometres from GPS locations between sampling sites.

### Microsatellite DNA analysis

All loci were tested for possible genotyping errors, the presence of null alleles, and allelic dropout using MICRO-CHECKER 2.2.3 software^23^. In this study, Arlequin 3.5^48^ was used to calculate the number of alleles (*N*_a_), mean observed heterozygosity (*H*_O_), mean expected heterozygosity (*H*_E_), and deviation from Hardy–Weinberg equilibrium (HWE) for each population. Allelic richness (*A*_R_) and inbreeding coefficient (*F*_IS_)^54^ were estimated using FSTAT version 2.9.3^55^. Population differentiation of *T. nanhaiensis* was studied by estimation of the *F*_ST_ and *R*_ST_ using Arlequin^48^ and AMOVA procedures as mitochondrial DNA data (mentioned above).

STRUCTURE v.2.2.3 was used to delineate the optimum number of homogenous groups of sampled individuals (K)^56^. A series of 10 independent runs for each K (ranging from 2 to 5) was performed using 100,000 iterations of the Markov chain Monte Carlo (MCMC) with a burn-in period of 20,000 chains. Structure Harvester Web 0.6.94 was used to determine that the optimum number of genetic clusters within the data were predicted using the delta K method and Evanno method^57^. GenAIEx version 6.503 was used to calculate allelic frequency^58^, and the R package “pheatmap” was used for data visualization and allele frequency heatmap construction. Moreover, to investigate the genetic relationship between populations, discriminant analysis of principal components (DAPC)^59^ was performed using the ‘“adegenet 2.0.1”’ package in R 3.2.2 software ^60^.

### Approximate Bayesian computation (ABC) analysis

The approximate Bayesian computation (ABC) method is incorporated into different historical and demographic evolutionary models that can be suited to complex problems that arise in scenarios in population genetics. To explore the possible historical demography of *T. nanhaiensis*, we used a coalescence analysis implemented in the program DIYABC v.2.0.4^61^ to obtain relevant and detailed information on the population history. We separately tested the five scenarios of possible demographic changes for *T. nanhaiensis* based on Cyt *b* and RyR3 sequences and microsatellite data (Fig. 8). The HKY was the best evolutionary model for our sequence dataset. The following scenarios were used: In scenario 1 (CON model), assumed constant population size of *T. nanhaiensis*; In scenario 2 (DEC model), assumed populations of *T. nanhaiensis* decline and had experienced a bottleneck event; In scenario 3 (INC model), assumed populations of *T. nanhaiensis* expansion recently; In scenario 4 (INCDEC model), assumed populations of *T. nanhaiensis* expanded followed by a single instantaneous decrease in population size; and in scenario 5 (DECINC model), assumed populations of *T. nanhaiensis* bottleneck followed by re‐expansion. To obtain robust results, a total of 3,000,000 simulations were performed as recommended by DIYABC for all five demographic scenarios (Fig. 8). We calculated all the summary statistics included in the software DIYABC. The intervals of the prior distribution of applied parameters were all 10–100,000, and those of mean mutation were 10^–9^–10^–7^. The best-supported scenario was obtained by the highest posterior probability, which were calculated by performing a polychotomous weighted logistic regression on 1% of the simulated datasets closest to the observed dataset^62^. Type I and II errors were calculated to evaluate the confidence of the selected scenario for each approach^62^.

## Supplementary Information

Supplementary Information 1.

Supplementary Information 2.

## Data Availability

The sequences of dataset were deposited in GenBank (accession numbers: OM303509-OM303789 for Cytb and OM388568-OM389129 for RYR3). Voucher specimens are housed at College of Fisheries, Guangdong Ocean University, Zhanjiang, China.
