# Supplementary material for: Genetic pattern and demographic history of cutlassfish (Trichiurus nanhaiensis) in South China Sea by the influence of Pleistocene climatic oscillations
Source: Sci Rep. 2022 Aug 30;12:14716. doi: 10.1038/s41598-022-18861-x (PMC9427976; doi:10.1038/s41598-022-18861-x)
Supplement: Supplementary file 1 — Supplementary Information 1. [file 41598_2022_18861_MOESM1_ESM.docx]

**Table S1** The distribution information of the shared haplotypes (Hm1-Hm181) (Cyt *b*). P indicate the number of the private haplotypes in each population

|  | ST | SZ | YJ | ZJ | BH | TD | SY |
| --- | --- | --- | --- | --- | --- | --- | --- |
| P | 32 | 17 | 20 | 20 | 25 | 17 | 28 |
| Hm1 |  |  |  |  | 1 |  |  |
| Hm2 |  |  |  |  | 1 |  |  |
| Hm3 |  |  |  |  | 1 |  |  |
| Hm4 |  |  |  |  | 1 |  |  |
| Hm5 |  |  |  |  | 1 |  |  |
| Hm6 |  |  |  |  | 1 |  |  |
| Hm7 |  |  |  |  | 1 |  |  |
| Hm8 | 4 | 2 | 1 | 2 | 1 |  | 1 |
| Hm9 |  | 1 | 1 |  | 1 |  |  |
| Hm10 |  |  |  |  | 1 |  |  |
| Hm11 |  |  |  |  | 1 |  |  |
| Hm12 |  |  |  |  | 1 |  | 1 |
| Hm13 |  |  |  |  | 1 |  |  |
| Hm14 |  |  |  |  | 1 |  |  |
| Hm15 |  |  |  |  | 1 |  |  |
| Hm16 |  |  |  |  | 1 |  |  |
| Hm17 |  |  |  |  | 1 |  |  |
| Hm18 | 2 | 1 | 3 | 1 | 4 |  | 1 |
| Hm19 |  |  |  |  | 1 |  |  |
| Hm20 | 1 |  | 2 |  | 1 | 1 | 2 |
| Hm21 |  |  |  |  | 1 |  |  |
| Hm22 |  |  |  |  | 1 |  |  |
| Hm23 |  |  |  |  | 1 |  |  |
| Hm24 |  |  |  |  | 1 |  |  |
| Hm25 |  | 2 |  | 1 | 1 |  | 1 |
| Hm26 |  |  |  |  | 1 |  |  |
| Hm27 |  | 1 | 1 | 1 | 3 | 1 | 2 |
| Hm28 | 6 | 3 |  |  | 1 |  | 1 |
| Hm29 |  |  |  |  | 1 |  |  |
| Hm30 |  |  |  |  | 1 |  |  |
| Hm31 |  |  |  |  | 1 |  |  |
| Hm32 |  |  | 1 |  | 1 |  |  |
| Hm33 |  |  |  |  | 1 |  |  |
| Hm34 |  |  | 1 | 1 | 1 |  |  |
| Hm35 |  |  |  |  | 1 |  |  |
| Hm36 | 1 |  |  |  |  |  |  |
| Hm37 | 1 |  |  |  |  |  |  |
| Hm38 | 1 |  |  |  |  |  |  |
| Hm39 | 1 |  | 1 |  |  |  |  |
| Hm40 | 1 |  |  |  |  |  |  |
| Hm41 | 1 |  | 1 |  |  |  |  |
| Hm42 | 1 |  |  |  |  |  |  |
| Hm43 | 4 | 2 |  |  |  | 2 | 1 |
| Hm44 | 1 |  |  |  |  |  |  |
| Hm45 | 1 |  |  |  |  |  |  |
| Hm46 | 1 |  |  |  |  |  |  |
| Hm47 | 1 |  |  |  |  |  |  |
| Hm48 | 2 |  |  |  |  |  |  |
| Hm49 | 1 |  |  |  |  |  |  |
| Hm50 | 4 | 3 |  | 2 |  |  | 4 |
| Hm51 | 3 |  | 1 |  |  |  |  |
| Hm52 | 1 |  |  |  |  |  |  |
| Hm53 | 1 |  |  |  |  |  |  |
| Hm54 | 1 |  |  |  |  |  |  |
| Hm55 | 1 |  |  |  |  |  |  |
| Hm56 | 1 |  |  |  |  |  |  |
| Hm57 | 1 | 1 | 1 |  |  |  |  |
| Hm58 | 1 | 1 |  |  |  |  |  |
| Hm59 | 1 | 2 |  |  |  |  |  |
| Hm60 | 1 |  |  |  |  |  |  |
| Hm61 | 1 |  |  |  |  |  |  |
| Hm62 | 1 |  |  |  |  |  |  |
| Hm63 | 1 |  |  |  |  |  |  |
| Hm64 | 1 |  |  |  |  |  |  |
| Hm65 | 1 |  |  |  |  |  |  |
| Hm66 | 1 |  |  |  |  |  |  |
| Hm67 | 1 |  |  |  |  |  |  |
| Hm68 | 1 |  |  |  |  |  |  |
| Hm69 | 1 |  |  |  |  |  |  |
| Hm70 | 1 |  |  |  |  |  |  |
| Hm71 | 1 |  |  |  |  |  |  |
| Hm72 | 1 |  | 1 |  |  |  |  |
| Hm73 | 1 |  |  |  |  |  |  |
| Hm74 | 1 |  |  |  |  |  |  |
| Hm75 | 1 |  |  |  |  |  |  |
| Hm76 | 1 |  |  |  |  |  |  |
| Hm77 |  |  |  |  |  |  | 1 |
| Hm78 |  |  |  |  |  |  | 1 |
| Hm79 |  |  |  |  |  |  | 1 |
| Hm80 |  |  |  |  |  |  | 1 |
| Hm81 |  | 1 |  | 1 |  | 2 | 4 |
| Hm82 |  |  |  |  |  |  | 1 |
| Hm83 |  |  |  |  |  |  | 1 |
| Hm84 |  |  |  |  |  |  | 1 |
| Hm85 |  |  |  |  |  |  | 1 |
| Hm86 |  |  |  |  |  |  | 1 |
| Hm87 |  |  |  |  |  |  | 1 |
| Hm88 |  |  |  |  |  |  | 1 |
| Hm89 |  |  |  |  |  |  | 1 |
| Hm90 |  |  |  |  |  |  | 1 |
| Hm91 |  |  |  |  |  |  | 1 |
| Hm92 |  |  |  |  |  |  | 1 |
| Hm93 |  |  |  |  |  |  | 1 |
| Hm94 |  |  |  |  |  |  | 1 |
| Hm95 |  |  |  | 1 |  |  | 1 |
| Hm96 |  |  |  |  |  |  | 1 |
| Hm97 |  |  |  |  |  |  | 1 |
| Hm98 |  |  |  |  |  |  | 1 |
| Hm99 |  |  |  |  |  |  | 1 |
| Hm100 |  |  |  |  |  |  | 1 |
| Hm101 |  |  |  |  |  |  | 1 |
| Hm102 |  | 2 |  |  |  | 1 | 1 |
| Hm103 |  |  |  |  |  |  | 1 |
| Hm104 |  |  |  |  |  |  | 1 |
| Hm105 |  |  |  |  |  |  | 1 |
| Hm106 |  |  |  |  |  |  | 1 |
| Hm107 |  |  |  |  |  |  | 1 |
| Hm108 |  | 1 |  |  |  |  |  |
| Hm109 |  | 1 |  |  |  |  |  |
| Hm110 |  | 1 |  |  |  |  |  |
| Hm111 |  | 1 |  |  |  |  |  |
| Hm112 |  | 2 |  |  |  |  |  |
| Hm113 |  | 1 |  |  |  |  |  |
| Hm114 |  | 1 |  |  |  |  |  |
| Hm115 |  | 1 |  |  |  |  |  |
| Hm116 |  | 1 |  |  |  |  |  |
| Hm117 |  | 1 |  |  |  |  |  |
| Hm118 |  | 1 |  |  |  |  |  |
| Hm119 |  | 1 |  |  |  |  |  |
| Hm120 |  | 1 |  |  |  |  |  |
| Hm121 |  | 1 |  |  |  |  |  |
| Hm122 |  | 1 |  |  |  |  |  |
| Hm123 |  | 1 |  |  |  |  |  |
| Hm124 |  | 1 |  |  |  |  |  |
| Hm125 |  |  |  |  |  | 1 |  |
| Hm126 |  |  |  |  |  | 1 |  |
| Hm127 |  |  |  |  |  | 1 |  |
| Hm128 |  |  |  |  |  | 1 |  |
| Hm129 |  |  |  |  |  | 1 |  |
| Hm130 |  |  |  |  |  | 1 |  |
| Hm131 |  |  |  |  |  | 1 |  |
| Hm132 |  |  |  |  |  | 1 |  |
| Hm133 |  |  |  |  |  | 1 |  |
| Hm134 |  |  |  |  |  | 1 |  |
| Hm135 |  |  |  |  |  | 1 |  |
| Hm136 |  |  |  |  |  | 2 |  |
| Hm137 |  |  |  |  |  | 1 |  |
| Hm138 |  |  |  |  |  | 1 |  |
| Hm139 |  |  |  |  |  | 1 |  |
| Hm140 |  |  |  |  |  | 1 |  |
| Hm141 |  |  |  |  |  | 1 |  |
| Hm142 |  |  |  | 1 |  |  |  |
| Hm143 |  |  |  | 1 |  |  |  |
| Hm144 |  |  |  | 1 |  |  |  |
| Hm145 |  |  |  | 1 |  |  |  |
| Hm146 |  |  |  | 1 |  |  |  |
| Hm147 |  |  |  | 1 |  |  |  |
| Hm148 |  |  |  | 1 |  |  |  |
| Hm149 |  |  |  | 1 |  |  |  |
| Hm150 |  |  |  | 1 |  |  |  |
| Hm151 |  |  |  | 1 |  |  |  |
| Hm152 |  |  |  | 1 |  |  |  |
| Hm153 |  |  |  | 1 |  |  |  |
| Hm154 |  |  |  | 1 |  |  |  |
| Hm155 |  |  |  | 1 |  |  |  |
| Hm156 |  |  |  | 1 |  |  |  |
| Hm157 |  |  |  | 1 |  |  |  |
| Hm158 |  |  |  | 1 |  |  |  |
| Hm159 |  |  |  | 1 |  |  |  |
| Hm160 |  |  |  | 1 |  |  |  |
| Hm161 |  |  |  | 1 |  |  |  |
| Hm162 |  |  | 1 |  |  |  |  |
| Hm163 |  |  | 1 |  |  |  |  |
| Hm164 |  |  | 1 |  |  |  |  |
| Hm165 |  |  | 1 |  |  |  |  |
| Hm166 |  |  | 1 |  |  |  |  |
| Hm167 |  |  | 1 |  |  |  |  |
| Hm168 |  |  | 1 |  |  |  |  |
| Hm169 |  |  | 1 |  |  |  |  |
| Hm170 |  |  | 1 |  |  |  |  |
| Hm171 |  |  | 1 |  |  |  |  |
| Hm172 |  |  | 1 |  |  |  |  |
| Hm173 |  |  | 1 |  |  |  |  |
| Hm174 |  |  | 1 |  |  |  |  |
| Hm175 |  |  | 1 |  |  |  |  |
| Hm176 |  |  | 1 |  |  |  |  |
| Hm177 |  |  | 1 |  |  |  |  |
| Hm178 |  |  | 1 |  |  |  |  |
| Hm179 |  |  | 1 |  |  |  |  |
| Hm180 |  |  | 1 |  |  |  |  |
| Hm181 |  |  | 1 |  |  |  |  |

**Table S2** The distribution information of the shared haplotypes (Hn1-Hn57) (RYR3). P indicate the number of the private haplotypes in each population

|  | ST | SZ | YJ | ZJ | BH | TD | SY |
| --- | --- | --- | --- | --- | --- | --- | --- |
| P | 9 | 4 | 2 | 4 | 6 | 4 | 14 |
| Hn1 | 52 | 44 | 33 | 31 | 39 | 26 | 34 |
| Hn2 | 33 | 12 | 24 | 6 | 16 | 10 | 23 |
| Hn3 |  | 2 |  |  | 1 | 1 |  |
| Hn4 | 1 |  |  | 2 | 1 |  | 1 |
| Hn5 | 1 |  | 1 | 1 | 1 | 1 | 3 |
| Hn6 | 6 | 2 | 1 | 1 | 2 | 3 | 1 |
| Hn7 |  |  |  |  | 1 |  |  |
| Hn8 | 19 | 14 | 5 | 10 | 10 | 1 | 9 |
| Hn9 |  |  |  |  | 1 |  |  |
| Hn10 |  |  |  |  | 1 |  |  |
| Hn11 |  |  |  |  | 1 |  |  |
| Hn12 |  |  |  |  | 2 |  |  |
| Hn13 |  |  |  |  | 2 |  |  |
| Hn14 | 2 |  |  |  |  |  |  |
| Hn15 | 1 |  |  |  |  |  |  |
| Hn16 | 1 |  |  |  |  |  |  |
| Hn17 | 1 |  |  |  |  |  |  |
| Hn18 | 1 |  |  |  |  |  |  |
| Hn19 | 1 |  |  |  |  |  |  |
| Hn20 | 2 |  |  | 2 |  |  | 2 |
| Hn21 | 2 |  |  |  |  |  |  |
| Hn22 | 2 |  |  |  |  |  |  |
| Hn23 | 1 |  |  |  |  |  |  |
| Hn24 |  | 1 |  |  |  |  | 1 |
| Hn25 |  |  |  |  |  |  | 1 |
| Hn26 |  |  |  |  |  |  | 1 |
| Hn27 |  |  |  |  |  |  | 1 |
| Hn28 |  |  |  |  |  |  | 2 |
| Hn29 |  |  |  |  |  |  | 2 |
| Hn30 |  |  |  | 1 |  |  | 1 |
| Hn31 |  | 1 |  | 1 |  |  | 1 |
| Hn32 |  |  | 2 |  |  |  | 2 |
| Hn33 |  |  |  |  |  |  | 1 |
| Hn34 |  |  |  |  |  |  | 1 |
| Hn35 |  |  |  |  |  |  | 1 |
| Hn36 |  |  |  |  |  |  | 2 |
| Hn37 |  |  |  |  |  |  | 2 |
| Hn38 |  |  |  |  |  |  | 1 |
| Hn39 |  |  |  |  |  |  | 1 |
| Hn40 |  |  |  |  |  |  | 1 |
| Hn41 |  |  |  |  |  |  | 1 |
| Hn42 |  | 1 |  |  |  |  |  |
| Hn43 |  | 1 |  |  |  |  |  |
| Hn44 |  | 1 |  |  |  |  |  |
| Hn45 |  | 1 |  |  |  |  |  |
| Hn46 |  |  | 2 |  |  | 1 |  |
| Hn47 |  |  | 2 |  |  |  |  |
| Hn48 |  |  | 1 |  |  |  |  |
| Hn49 |  |  | 1 |  |  | 3 |  |
| Hn50 |  |  |  | 2 |  |  |  |
| Hn51 |  |  |  | 1 |  |  |  |
| Hn52 |  |  |  | 1 |  |  |  |
| Hn53 |  |  |  | 1 |  |  |  |
| Hn54 |  |  |  |  |  | 1 |  |
| Hn55 |  |  |  |  |  | 1 |  |
| Hn56 |  |  |  |  |  | 1 |  |
| Hn57 |  |  |  |  |  | 1 |  |

**Table S3** Matrix of pairwise *F*_ST_ based on RYR 3 (below diagonal) and Cyt *b* (above diagonal). Refer to Table 1 for the abbreviations of localities

|  | ST | SZ | YJ | ZJ | BH | TD | SY |
| --- | --- | --- | --- | --- | --- | --- | --- |
| ST |  | 0.004 | 0.034^**^ | 0.040^***^ | 0.034^**^ | 0.076^***^ | 0.030^**^ |
| SZ | 0.016 |  | 0.018^**^ | 0.027 | 0.007 | 0.084 | 0.015 |
| YJ | 0.004 | 0.053^*^ |  | 0.006 | -0.011 | 0.013 | -0.011 |
| ZJ | 0.025^*^ | 0.005 | 0.060^***^ |  | 0.009 | 0.037 | -0.009 |
| BH | 2.80*10^-4^ | 0.005 | 0.014 | 0.018 |  | 0.055^*^ | -0.005 |
| TD | 0.021^**^ | 0.056^**^ | 0.012 | 0.050^**^ | 0.020 |  | 0.022 |
| SY | 0.002^**^ | 0.021^*^ | 0.005 | 0.017 | 0.006 | 0.021 |  |

**Table S4** Characteristics and genetic diversity indices for thirteen microsatellite loci in *Trichiurus nanhaiensis.*

| Locus | N_a_^a^ | A_R_^b^ | H_O_^c^ | H_E_^d^ | F_ST_ | R_ST_ | F_IS_ |
| --- | --- | --- | --- | --- | --- | --- | --- |
| Tna1 | 7 | 5.523 | 0.244 | 0.343 | 0.0148 | 0.012 | 0.294 |
| Tna2 | 7 | 6.177 | 0.741 | 0.751 | -0.0011 | 0.001 | 0.013 |
| Tna3 | 3 | 2.771 | 0.336 | 0.409 | 0.0032 | 0.025 | 0.167 |
| Tna13 | 8 | 7.118 | 0.738 | 0.775 | -0.0001 | -0.006 | 0.049 |
| Tna14 | 12 | 10.368 | 0.759 | 0.814 | 0.0023 | 0.031 | 0.068 |
| Tna16 | 27 | 22.406 | 0.672 | 0.951 | 0.0002 | -0.004 | 0.297 |
| Tna41 | 6 | 5.344 | 0.606 | 0.642 | -0.0025 | 0.041 | 0.058 |
| Tna42 | 7 | 6.119 | 0.668 | 0.654 | 0.0056 | 0.003 | -0.017 |
| Tna44 | 4 | 3.736 | 0.556 | 0.611 | 0.0093 | 0.013 | 0.103 |
| Tna45 | 8 | 6.669 | 0.632 | 0.705 | 0.0020 | -0.009 | 0.105 |
| Tna46 | 8 | 6.988 | 0.669 | 0.744 | 0.0016 | 0.015 | 0.103 |
| Tna48 | 13 | 11.595 | 0.619 | 0.865 | 0.0007 | 0.014 | 0.286 |
| Tna49 | 8 | 6.922 | 0.482 | 0.745 | -0.0006 | -0.009 | 0.354 |
| Mean | 9 | 7.826 | 0.594 | 0.693 | 0.0027 | 0.010 | 0.145 |

^a^ Number of alleles; ^b^ Mean allelic richness; ^c^ Mean observed heterozygosity; ^d^ Mean expected heterozygosity.

**Table S5** Matrix of painrwise *F*_ST_ (below diagonal) and R_ST_ (above diagonal) among seven populations based on microsatellite DNA in *Trichiurus nanhaiensis.*

|  | ST | SZ | YJ | ZJ | BH | TD | SY |
| --- | --- | --- | --- | --- | --- | --- | --- |
| ST |  | -0.006 | 0.011 | 0.028^*^ | -0.001 | 0.004 | 0.001 |
| SZ | 0.003 |  | 0.003 | 0.026 | 0.001 | -0.014 | 0.006 |
| YJ | -0.001 | -0.001 |  | 0.007 | 0.017 | 0.013 | 0.003 |
| ZJ | -0.001 | 0.003 | -0.002 |  | 0.008 | 0.040 | 0.009 |
| BH | 0.003 | 0.007^*^ | 0.001 | 0.001 |  | 0.015 | 0.002 |
| TD | 0.012^***^ | 0.004 | 0.009 | 0.010^*^ | 0.010^*^ |  | 0.008 |
| SY | 0.002 | 0.007^*^ | 0.003 | -0.001 | 0.002 | 0.014^**^ |  |

^*^P<0.05, ^**^P<0.01, ^***^P<0.001 for indices of population differentiation.

**Table S6** The results of Structure analysis from K=1 to 5

| K | Stdev LnP(K) | Ln’(K) | \|Ln’’(K)\| | Delta K |
| --- | --- | --- | --- | --- |
| 1 | 0.792 | - | - | - |
| 2 | 27.976 | -172.360 | 168.880 | 6.036 |
| 3 | 85.422 | -3.480 | 111.380 | 1.301 |
| 4 | 41.281 | 107.900 | 86.120 | 2.086 |
| 5 | 31.630 | 21.780 | - | - |

**Figure captions**

Fig. S1. Phylogenetic trees reconstructed from mitochondrial sequences of the RyR3 gene in *Trichiurus nanhaiensis*. The values above the branches are bootstrap values for the NJ, BI, and ML analyses.

Fig. S2. The minimum‐spanning haplotype network inferred from the nuclear RyR3 gene of *Trichiurus nanhaiensis* from seven geographic samples in mainland China. Each circle indicates each haplotype, and the size of each circle is related to its haplotype distribution frequency. Each color in haplotype network circles represents each geographic sample.

Fig. S3. Mismatch distributions of *Trichiurus nanhaiensis* based on the mitochondrial cyt *b* gene.


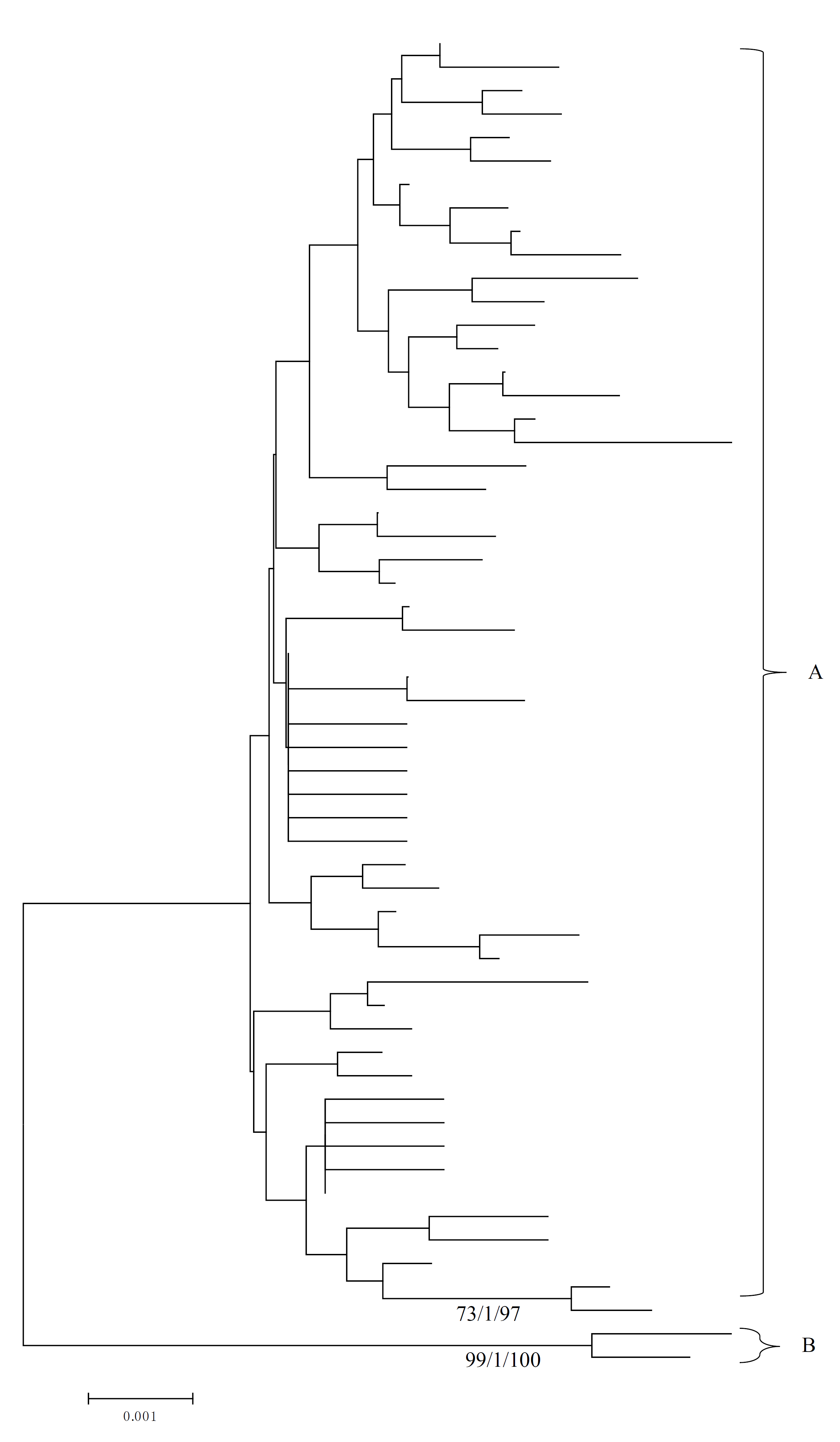


Fig. S1.


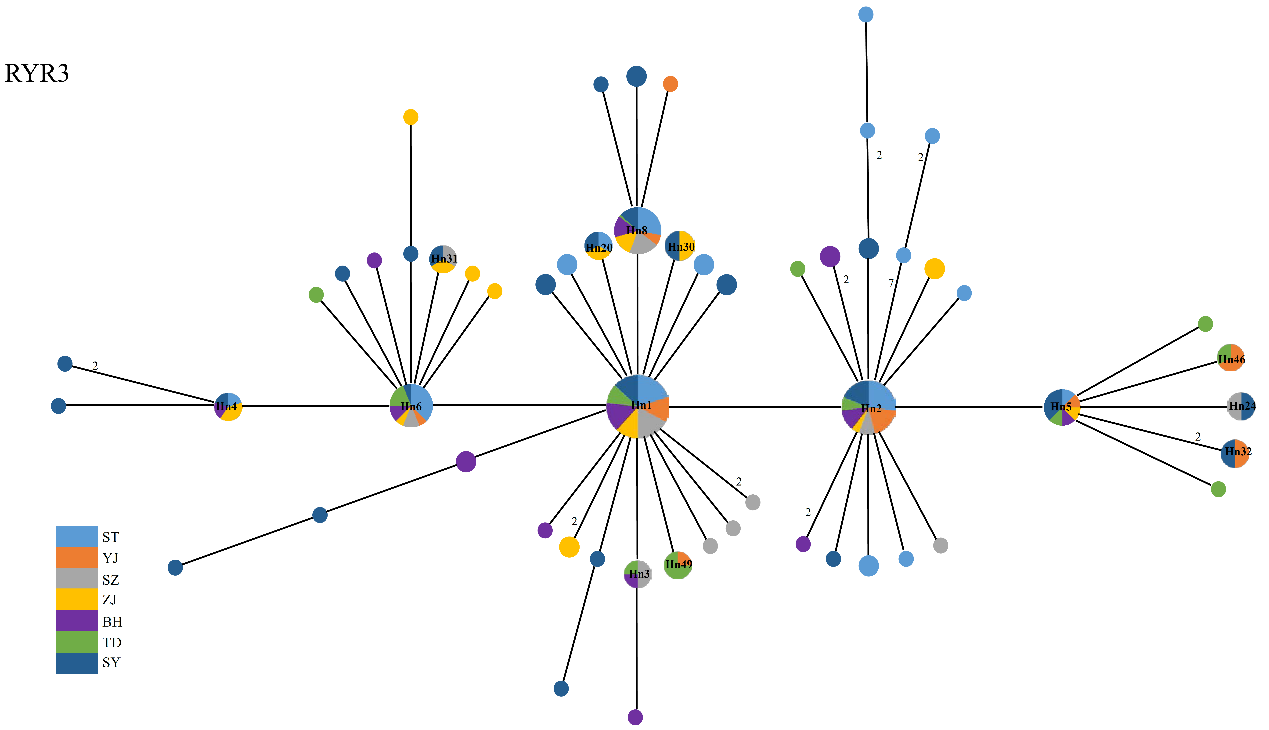


Fig. S2


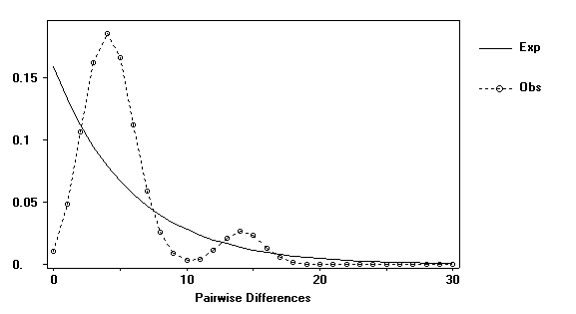


Fig. S3.
